# Proteomic response of *Turicibacter bilis* MMM721 to chicken bile and its bile acids

**DOI:** 10.1186/s13104-022-06127-8

**Published:** 2022-07-02

**Authors:** Joel J. Maki, John D. Lippolis, Torey Looft

**Affiliations:** 1 Food Safety and Enteric Pathogens Research Unit, National Animal Disease Center, Agricultural Research Service, United States Department of Agriculture, Ames, IA 50010 USA; 2 Interdepartmental Microbiology Graduate Program, Iowa State University, Ames, IA 50011 USA; 3 Ruminant Diseases and Immunology Research UnitAgricultural Research ServiceDepartment of Agriculture, National Animal Disease Center, Ames, IA 50010 USA

**Keywords:** *Turicibacterbilis*, Bile Acids, Chicken, Proteomics

## Abstract

**Objective:**

Bile and its individual components, mainly bile acids, are important for digestion and drive bacterial community dynamics in the upper gastrointestinal tract of chickens. However, specific responses to bile acids have been characterized in only a few commensal bacteria, and it is unclear how other members of the microbiota respond to biliary stress. Here, we used label-free LC–MS/MS to assess the proteomic response of a common inhabitant of the chicken small intestine, *Turicibacter bilis* MMM721, to 24 h of growth in anaerobic growth media supplemented with 0.1% whole chicken bile, 0.1% taurochenodeoxycholic acid (TCDCA), or 0.1% taurocholic acid (TCA).

**Results:**

Seventy, 46, and 10 differentially expressed proteins were identified in *Turicibacter bilis* MMM721 cultured with supplements of chicken bile, TCDCA, and TCA, respectively, when compared to unsupplemented controls. Many differentially expressed proteins were predicted to be involved in ribosomal processes, post-translational modifications and chaperones, and modifications to the cell surface. Ultimately, the *T. bilis* MMM721 response to whole bile and bile acids is complex and may relate to adaptations for small intestine colonization, with numerous proteins from a variety of functional categories being impacted.

**Supplementary Information:**

The online version contains supplementary material available at 10.1186/s13104-022-06127-8.

## Introduction

Bile is critical for digestion and interacts with microbes within the gastrointestinal tract (GIT). The major solutes in bile are bile acids (BAs), traditionally considered to be antimicrobial compounds [1–4]. BAs greatly impact bacteria colonizing the small intestine, where high concentrations lead to membrane disruption, DNA damage, and protein misfolding [5–10]. In chickens, the primary BAs are ~ 15% taurocholic acid (TCA) and ~ 85% taurochenodeoxycholic acid (TCDCA) [4, 11–13].

*Turicibacter* are anaerobic commensals of the upper GIT of numerous species [14–18]. Recently, *Turicibacter bilis* MMM721 (henceforth referred to as MMM721) was isolated from chicken eggshells during a study of bacterial succession and is capable of colonizing the small intestine where it likely interacts with bile and BAs [18].

Despite the importance of bacteria-BA interactions, few studies have measured the proteomic response of individual gut bacteria to bile and BAs. The ubiquitous nature of *Turicibacter* spp. in the upper intestinal tract of vertebrate species suggests the genus has mechanisms through which it can tolerate the stress induced by BAs. Here, we use survival assays and LC–MS/MS proteomics to interrogate the response of MMM721 to chicken bile and its BA components: TCDCA and TCA.

## Main text

### Materials and methods

#### Growth conditions

The isolation of MMM721 has been described previously [19]. MMM721 was maintained anaerobically on Brain Heart Infusion (Difco) agar (pH 7.0) supplemented with 1.0% (v/v) glycerol and 1.1% (w/v) sodium DL-lactate (BHIGL) at 42 °C as previously described [19]. MMM721 was grown for 24 h on BHIGL prior to use in experiments. Plates were scraped and inoculated into BHIGL broth, BHIGL broth with 0.1% (v/v) whole chicken bile (BHIGL + Bile), 0.1% (w/v) TCDCA (BHIGL + TCDCA), or 0.1% (w/v) TCA (BHIGL + TCA). Whole bile was collected from the gallbladder of euthanized white leghorn chickens at the National Animal Disease Center (Ames, IA). TCA and TCDCA were purchased from Sigma-Aldrich. Cultures were incubated anaerobically (85% N_2_, 5% CO_2_, 10% H_2_) for 24 h at 42 °C*.*

Growth curves for BHIGL broth with 1.0% BHIGL + Bile, 0.1% BHIGL + Bile, 0.1% BHIGL + TCDCA, and 0.1% (w/v) BHIGL + TCA were conducted with a Bioscreen C automated turbidity reader which measures the optical density (OD_600_) hourly. Growth curves were conducting with nine technical replicates.

For growth assays, colony forming units (CFU)/mL were determined on BHIGL immediately after inoculation using previously described methods [20]. After 24 h, cultures were plated on BHIGL agar and incubated for 48 h to determine CFU/mL. Three biological replicates were conducted for each condition tested with two technical replicates each.

For proteomic analysis, cells from broth cultures described above, were harvested via centrifugation. Pellets were washed twice with 1∙phosphate-buffered saline (PBS) and resuspended in 3 mL of cold 50 mM ammonium bicarbonate buffer (pH 8.0) prior to freezing at -80 °C. Three biological replicates of each strain were conducted for each condition tested. All centrifugation steps were 4000×*g* for 20 m at 4 °C.

#### Turicibacter bilis MMM721 genome annotation

Genomic features for MMM721 have been described previously [19]. MMM721 was annotated with Prokka v1.14.4 [21]. Prokka-identified open reading frames (ORFs) were submitted to EggNOG-Mapper (http://eggnog-mapper.embl.de) for functional assignments [22].

#### LC–MS/MS sample preparation

Cell pellets were thawed and distributed into 2×1.5 ml screw-cap bead-beating tubes filled with 300 μl of 0.1 mm zirconia/silica beads (BioSpec Products, Inc.). Tubes were placed on a Vortex-Genie with tube-holder attachment (USA Scientific) and agitated at maximum speed for 3 min followed by 5 min on ice. This was repeated for 5 cycles, after which samples were spun for 10 m (16000 g) at 4 °C. Supernatant was removed and protein concentrations were determined using a Bradford protein assay (Bio-Rad). Samples with low protein concentrations were concentrated with Amicon Ultra-15 (10 kDa cut-off) Centrifugal Filter Units (Millipore). Trypsin/Lys-C Mix, Mass Spec Grade (Promega) was used to digest 40 μg of protein from each sample. Samples were then desalted, dried, and resuspended using previously described methods prior to LC–MS/MS [23]. All preparation steps were conducted according to the manufacturer’s instructions.

### Data analysis

Raw data files were processed in MaxQuant v1.6.7.0 and Perseus v1.6.7.0 using previously described parameters [23–25]. The Prokka-annotated *Turicibacter bilis* MMM721 genome was used to construct a protein database for proteomic analysis. Protein expression levels were transformed to log2 quantities. Peptides were considered significantly differentially expressed peptides (DEPs) if they had a false discovery rate q < 0.05 and a log_2_ difference of greater than 0.6 or less than −0.6. Statistical analyses and figure generation were conducted in R [26, 27].

## Results

### Preliminary growth curve

Growth was observed in 0.1% bile but not in 1.0% bile. Growth curves for MMM721 in BHIGL + Bile, BHIGL + TCDCA, BHIGL + TCA, and BHIGL suggested the strain reached stationary phase somewhere between 12–20 h after inoculation (Additional File 1). An incubation time of 24 h was chosen for the survival assay and proteomics to allow time for MMM721 to mount a response to supplemented bile and BAs.

### Reduced MMM721 growth in the presence of bile and TCDCA

**Fig. 1:**
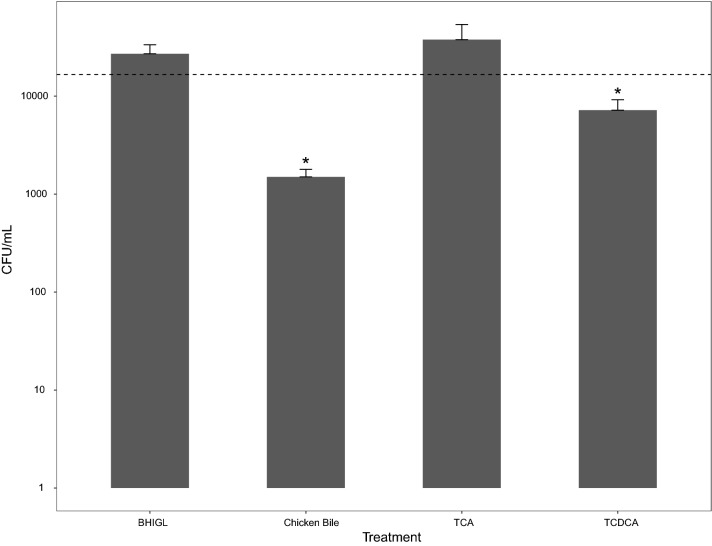
*Turicibacter bilis* MMM721 growth in BHIGL, BHIGL + Bile, BHIGL + TCA, and BHIGL + TCDCA. Bacterial CFU/mL after 24 h were determined on BHIGL agar. The dashed line denotes the mean CFU/mL at inoculation (hour 0). *differences between the initial CFU/mL immediately after inoculation vs. CFU/mL after 24 h as determined by the t.test function in R (p.adjusted < 0.05). Error bars represent standard error of the mean

Small increases in MMM721 CFU/mL were observed in un-supplemented BHIGL (2.9 × 10^4^ CFU/mL) and BHIGL + TCA (4.2 × 10^4^ CFU/mL). Incubation in BHIGL + Bile (1.5 × 10^3^ CFU/mL) or BHIGL + TCDCA (7.7 × 10^3^ CFU/mL) elicited significant (p.adj < 0.05) decreases in vegetative cells after 24 h (Fig. 1).

### Proteomic data confirm differential response of MMM721 to bile and TCDCA

**Fig. 2:**
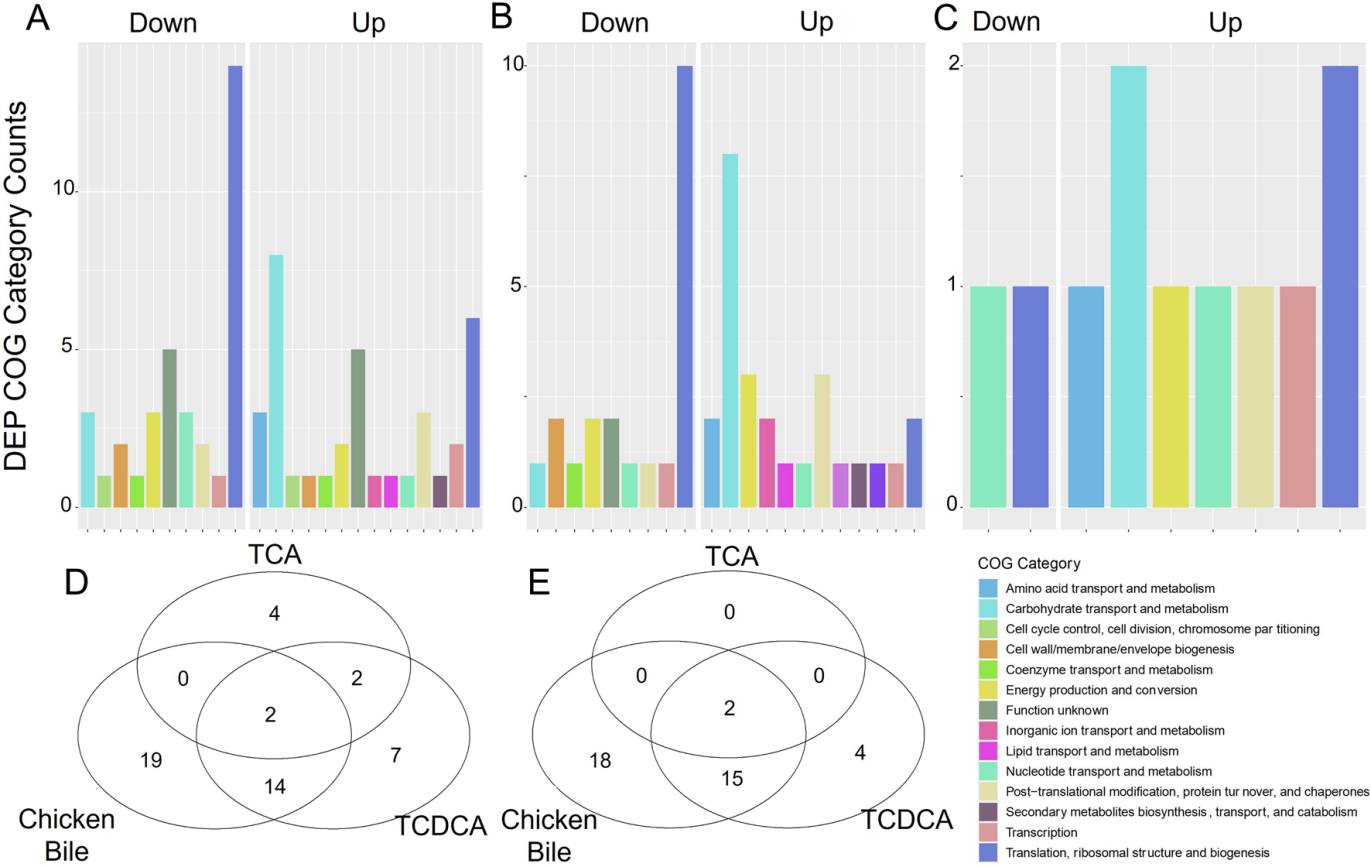
*T. bilis* MMM721 DEPs in BHIGL + Bile, BHIGL + TCDCA, and BHIGL + TCA compared to BHIGL only control. Barcharts of COG category counts of DEPs after 24 h of incubation in **A** BHIGL + BHIGL, **B** BHIGL + TCDCA, and **C** BHIGL + TCA compared to a BHIGL-only control. COG category assignments and protein descriptions were provided by EggNOG-mapper. Venn diagrams of shared and unique DEPs that were **D** up-regulated and **E** down-regulated after 24 h in BHIGL + Bile, BHIGL + TCDCA, and BHIGL + TCA as compared to BHIGL only

Label-free LC–MS/MS was used to identify and quantitate DEPs in the supplemented media relative to BHIGL alone. Of the 70 DEPs identified in BHIGL + Bile compared to BHIGL, 35 were increased and 35 were reduced (Fig. 2A, Table 1). Clusters of Orthologous Group (COG) category G (carbohydrate transport and metabolism) had the most increased DEPs (Fig. 2A). COG category J (translation, ribosomal structure, and biogenesis) had the most decreased DEPs in MMM721 grown in BHIGL + Bile, though several COG J DEPs were also highly expressed (Table 1).

**Table 1:** Table of DEPs in *T. bilis* MMM721 DEPs in BHIGL + Bile, BHIGL + TCDCA, and BHIGL + TCA compared to BHIGL only control. Bolded values were DEPs in BHIGL + Bile, BHIGL + TCDCA, or BHIGL + TCA. The COG category assignments and protein descriptions were provided by EggNOG-mapper

Cog Category	Prokka_ID	BHIGL + Bile Log2 Difference	BHIGL + TCDCA Log2 Difference	BHIGL + TCA Log2 Difference	Description
C: Energy production and conversion	ONGNNMAF_01437	3.4	2.0	−0.2	F(1)F(0) ATP synthase produces ATP from ADP in the presence of a proton or sodium gradient. F-type ATPases consist of two structural domains, F[1] containing the extramembraneous catalytic core and F(0) containing the membrane proton channel, linked together by a central stalk and a peripheral stalk. During catalysis, ATP synthesis in the catalytic domain of F is coupled via a rotary mechanism of the central stalk subunits to proton translocation
	ONGNNMAF_01709	0.8	1.5	1.2	Activation of pyruvate formate-lyase under anaerobic conditions by generation of an organic free radical, using S- adenosylmethionine and reduced flavodoxin as cosubstrates to produce 5'-deoxy-adenosine
	ONGNNMAF_01722	1.1	1.7	0.4	Nitroreductase family
	ONGNNMAF_01745	1.1	0.6	3.7	Dehydrogenase
	ONGNNMAF_01769	−2.5	−2.1	−0.8	Belongs to the nitrite and sulfite reductase 4Fe-4S domain family
	ONGNNMAF_02115	−3.1	−3.3	−0.7	Belongs to the class-II pyridine nucleotide-disulfide oxidoreductase family
	ONGNNMAF_02459	−1.2	−0.9	0.6	SUF system FeS assembly protein, NifU family
	ONGNNMAF_02607	1.4	0.8	−0.5	belongs to the iron- containing alcohol dehydrogenase family
D: Cell cycle control, cell division, chromosome partitioning	ONGNNMAF_00642	1.6	0.6	0.1	Involved in protein export. Acts as a chaperone by maintaining the newly synthesized protein in an open conformation. Functions as a peptidyl-prolyl cis–trans isomerase
	ONGNNMAF_01411	−1.2	−0.1	0.9	Peptidase family M23
E: Amino acid transport and metabolism	ONGNNMAF_00140	1.9	1.4	0.2	Cleaves the N-terminal amino acid of tripeptides
	ONGNNMAF_02259	3.8	4.4	3.2	Peptidase dimerisation domain
	ONGNNMAF_02452	1.4	0.0	−0.7	Aminopeptidase P, N-terminal domain
F: Nucleotide transport and metabolism	ONGNNMAF_00874	−0.8	−0.7	0.8	Belongs to the cytidylate kinase family. Type 1 subfamily
	ONGNNMAF_01593	−2.9	−3.0	−2.0	Catalyzes the synthesis of GMP from XMP
	ONGNNMAF_01630	−0.9	−0.3	−0.6	Belongs to the purine pyrimidine phosphoribosyltransferase family
	ONGNNMAF_01948	0.9	1.2	1.0	Belongs to the d-alanine–d-alanine ligase family
G: Carbohydrate transport and metabolism	ONGNNMAF_00074	−0.1	0.7	1.3	Psort location Cytoplasmic, score 9.98
	ONGNNMAF_00350	2.2	1.7	1.1	Psort location Cytoplasmic, score
	ONGNNMAF_00457	2.1	1.9	0.0	M42 glutamyl aminopeptidase
	ONGNNMAF_00502	3.0	1.3	1.1	Belongs to the glycosyl hydrolase 13 family
	ONGNNMAF_01096	−0.8	−0.7	−0.3	PTS system, glucose subfamily, IIA
	ONGNNMAF_01444	1.0	0.8	0.6	Psort location Cytoplasmic, score 8.87
	ONGNNMAF_01872	−1.4	−1.3	−0.9	Fructose-1,6-bisphosphatase
	ONGNNMAF_01935	−1.6	−0.9	0.5	Catalyzes the conversion of glucosamine-6-phosphate to glucosamine-1-phosphate
	ONGNNMAF_02092	1.2	1.0	0.7	Psort location Cytoplasmic, score
	ONGNNMAF_02192	1.6	2.5	1.3	Domain of unknown function (DUF5110)
	ONGNNMAF_02194	2.5	3.4	1.8	Melibiase
	ONGNNMAF_02435	1.0	1.9	0.8	Bacterial extracellular solute-binding protein
	ONGNNMAF_02481	2.0	3.7	0.9	Bacterial extracellular solute-binding protein
H: Coenzyme transport and metabolism	ONGNNMAF_00285	−0.7	−2.6	−1.0	6-pyruvoyl tetrahydropterin synthase
	ONGNNMAF_02112	−0.6	0.6	0.4	Catalyzes the ATP- as well as the pyrophosphate- dependent phosphorylation of a specific serine residue in HPr, a phosphocarrier protein of the phosphoenolpyruvate-dependent sugar phosphotransferase system (PTS). HprK P also catalyzes the pyrophosphate-producing, inorganic phosphate-dependent dephosphorylation (phosphorolysis) of seryl-phosphorylated HPr (P- Ser-HPr). The two antagonistic activities of HprK P are regulated by several intracellular metabolites, which change their concentration in response to the absence or presence of rapidly metabolisable carbon sources (glucose, fructose, etc.) in the growth medium. Therefore, by controlling the phosphorylation state of HPr, HPrK P is a sensor enzyme that plays a major role in the regulation of carbon metabolism and sugar transport it mediates carbon catabolite repression (CCR), and regulates PTS-catalyzed carbohydrate uptake and inducer exclusion
	ONGNNMAF_02537	2.9	1.9	2.3	Catalyzes the synthesis of ADP-glucose, a sugar donor used in elongation reactions on alpha-glucans
I: Lipid transport and metabolism	ONGNNMAF_00468	1.5	0.9	0.9	Dehydrogenase
J: Translation, ribosomal structure and biogenesis	ONGNNMAF_00551	0.8	0.7	1.8	Catalyzes the attachment of alanine to tRNA(Ala) in a two-step reaction alanine is first activated by ATP to form Ala- AMP and then transferred to the acceptor end of tRNA(Ala). Also edits incorrectly charged Ser-tRNA(Ala) and Gly-tRNA(Ala) via its editing domain
	ONGNNMAF_00686	−1.5	−1.3	−0.7	Belongs to the universal ribosomal protein uS2 family
	ONGNNMAF_00699	3.3	3.3	1.9	This protein is located at the 30S-50S ribosomal subunit interface and may play a role in the structure and function of the aminoacyl-tRNA binding site
	ONGNNMAF_00877	−1.2	−1.5	−0.3	Belongs to the pseudouridine synthase RsuA family
	ONGNNMAF_01070	−1.1	−0.1	0.0	amino acids such as threonine, to avoid such errors, it has a posttransfer editing activity that hydrolyzes mischarged Thr-tRNA(Val) in a tRNA-dependent manner
	ONGNNMAF_01175	−1.4	0.1	0.5	Belongs to the class-I aminoacyl-tRNA synthetase family
	ONGNNMAF_01458	−1.3	−1.3	0.2	ATPase that binds to both the 70S ribosome and the 50S ribosomal subunit in a nucleotide-independent manner
	ONGNNMAF_01634	3.7	1.5	0.8	RNA binding protein, contains ribosomal protein S1 domain
	ONGNNMAF_01830	−0.8	−0.6	0.3	Arginyl-tRNA synthetase
	ONGNNMAF_01892	−1.8	−1.5	−0.2	Forms part of the ribosomal stalk which helps the ribosome interact with GTP-bound translation factors
	ONGNNMAF_01894	1.7	1.1	−0.2	Forms part of the ribosomal stalk, playing a central role in the interaction of the ribosome with GTP-bound translation factors
	ONGNNMAF_01901	−1.0	−0.6	0.6	Catalyzes the GTP-dependent ribosomal translocation step during translation elongation. During this step, the ribosome changes from the pre-translocational (PRE) to the post- translocational (POST) state as the newly formed A-site-bound peptidyl-tRNA and P-site-bound deacylated tRNA move to the P and E sites, respectively. Catalyzes the coordinated movement of the two tRNA molecules, the mRNA and conformational changes in the ribosome
	ONGNNMAF_01904	−0.3	−1.1	−0.5	One of the primary rRNA binding proteins, it binds directly near the 3’-end of the 23S rRNA, where it nucleates assembly of the 50S subunit
	ONGNNMAF_01909	3.7	0.4	−0.1	The globular domain of the protein is located near the polypeptide exit tunnel on the outside of the subunit, while an extended beta-hairpin is found that lines the wall of the exit tunnel in the center of the 70S ribosome
	ONGNNMAF_01910	−1.3	−1.2	−0.7	Binds the lower part of the 30S subunit head. Binds mRNA in the 70S ribosome, positioning it for translation
	ONGNNMAF_01911	−2.3	−3.0	−0.7	Binds 23S rRNA and is also seen to make contacts with the A and possibly P site tRNAs
	ONGNNMAF_01913	−1.8	−2.0	−0.5	One of the primary rRNA binding proteins, it binds specifically to the 5’-end of 16S ribosomal RNA
	ONGNNMAF_01914	−2.1	−2.5	−1.5	Binds to 23S rRNA. Forms part of two intersubunit bridges in the 70S ribosome
	ONGNNMAF_01915	−1.1	−2.0	−1.3	One of the proteins that surrounds the polypeptide exit tunnel on the outside of the subunit
	ONGNNMAF_01920	2.5	−0.4	−1.3	This is one of the proteins that binds and probably mediates the attachment of the 5S RNA into the large ribosomal subunit, where it forms part of the central protuberance
	ONGNNMAF_01923	−1	−1.3	−0.7	binds to the 23S rRNA
	ONGNNMAF_02102	−1.6	−1.1	0.6	tRNA synthetases class I (E and Q), anti-codon binding domain
	ONGNNMAF_02594	0	1.3	1.8	Psort location Cytoplasmic, score
	ONGNNMAF_02602	5.6	0.6	0.7	One of the primary rRNA binding proteins, it binds directly to 16S rRNA where it nucleates assembly of the body of the 30S subunit
	ONGNNMAF_02642	2	0.9	1.6	Removes the formyl group from the N-terminal Met of newly synthesized proteins. Requires at least a dipeptide for an efficient rate of reaction. N-terminal L-methionine is a prerequisite for activity but the enzyme has broad specificity at other positions
K: Transcription	ONGNNMAF_00074	−0.1	0.7	1.3	Psort location Cytoplasmic, score 9.98
	ONGNNMAF_00373	−0.8	−2.1	−1.9	Cold-shock DNA-binding domain protein
	ONGNNMAF_00603	1.4	1.3	0.8	Negative regulator of class I heat shock genes (grpE- dnaK-dnaJ and groELS operons). Prevents heat-shock induction of these operons
	ONGNNMAF_01042	2.3	1.6	−0.9	Cold-shock DNA-binding domain protein
	ONGNNMAF_02616	−2.1	NA	0.1	Psort location Cytoplasmic, score
L: Replication, recombination and repair	ONGNNMAF_01805	0.0	2.4	1.2	A type II topoisomerase that negatively supercoils closed circular double-stranded (ds) DNA in an ATP-dependent manner to modulate DNA topology and maintain chromosomes in an underwound state. Negative supercoiling favors strand separation, and DNA replication, transcription, recombination and repair, all of which involve strand separation. Also able to catalyze the interconversion of other topological isomers of dsDNA rings, including catenanes and knotted rings. Type II topoisomerases break and join 2 DNA strands simultaneously in an ATP-dependent manner
M: Cell wall/membrane/ envelope biogenesis	ONGNNMAF_00060	−1.2	−1.0	0.7	Choline/ethanolamine kinase
	ONGNNMAF_00349	2.2	1.7	0.6	FMN-binding domain protein
	ONGNNMAF_01300	−1.7	−1.8	−0.5	Catalyzes the reduction of dTDP-6-deoxy-L-lyxo-4- hexulose to yield dTDP-L-rhamnose
O: Post-translational modification, protein turnover, and chaperones	ONGNNMAF_00204	1.8	1.4	1.2	Molecular chaperone. Has ATPase activity
	ONGNNMAF_00641	−0.4	−0.1	1.4	ATP-dependent Clp protease ATP-binding subunit ClpX
	ONGNNMAF_00848	−1.1	−1.7	−0.6	PPIases accelerate the folding of proteins. It catalyzes the cis–trans isomerization of proline imidic peptide bonds in oligopeptides
	ONGNNMAF_01227	1.6	1.4	0.2	Peroxiredoxin
	ONGNNMAF_01975	−0.9	−0.4	0.0	peptidylprolyl isomerase
	ONGNNMAF_02078	0.6	1.2	0.5	Prevents misfolding and promotes the refolding and proper assembly of unfolded polypeptides generated under stress conditions
	ONGNNMAF_02457	1.3	1.7	1.1	SufB sufD domain protein
P: Inorganic ion transport and metabolism	ONGNNMAF_01712	1.5	1.9	0.8	Part of the ABC transporter complex MetNIQ involved in methionine import. Responsible for energy coupling to the transport system
	ONGNNMAF_02637	1.8	2.2	−0.2	TrkA N-terminal domain protein
Q: Secondary metabolites biosynthesis, transport, and catabolism	ONGNNMAF_00468	1.5	0.9	0.9	Dehydrogenase
S: Function unknown	ONGNNMAF_00252	−2.0	−2.0	−0.7	Hypothetical protein
	ONGNNMAF_00275	−0.9	−0.4	0.1	Hypothetical protein
	ONGNNMAF_00984	−2.6	−2.2	−0.3	Pyridine nucleotide-disulphide oxidoreductase, dimerisation domain
	ONGNNMAF_01156	2.9	1.3	0.6	CYTH
	ONGNNMAF_01493	−1.5	−1.1	−0.2	Hypothetical protein
	ONGNNMAF_01796	1.7	0.7	0.6	Psort location Cytoplasmic, score
	ONGNNMAF_01808	2.0	1.6	0.2	S4 domain protein YaaA
	ONGNNMAF_01956	1.5	0.8	−0.6	An RNase that has 5'-3' exonuclease and possibly endonuclease activity. Involved in maturation of rRNA and in some organisms also mRNA maturation and or decay
	ONGNNMAF_02132	1.0	1.2	0.9	Domain of unknown function (DUF4358)
	ONGNNMAF_02639	−1.1	−0.9	−0.3	An RNase that has 5'-3' exonuclease and possibly endonuclease activity. Involved in maturation of rRNA and in some organisms also mRNA maturation and or decay
T: Signal transduction mechanisms	ONGNNMAF_02581	1.1	2.0	1.5	Psort location Cytoplasmic, score
U: Intracellular trafficking, secretion, and vesicular transport					
V: Defense mechanisms					

BHIGL + TCDCA displayed 46 DEPs (25 increased/21 reduced) compared to BHIGL, with COG G having the most increased DEPs and COG J having the most decreased DEPs (Table 1, Fig. 2B).

Ten DEPs (8 increased/2 reduced) were identified when comparing BHIGL + TCA to BHIGL. Both COG categories G and J had multiple proteins increased in BHIGL + TCA (Fig. 2C). Several of these proteins were undescribed cytoplasmic proteins (Table 1). A dehydrogenase (ONGNNMAF_01745) belonging to COG C (Energy production and conversion) and CplX (ONGNNMAF_00641), an ATPase belonging to COG O, were both increased in BHIGL + TCA.

Two DEPs were increased in all treatments, peptidase dimerization protein (ONGNNMAF_02259) and d-alanine- d-alanine ligase (ONGNNMAF_01948) (Fig. 2D, Table 1). GMP synthase (ONGNNMAF_01593) and an intersubunit bridge protein (ONGNNMAF_01914) were decreased across all treatments (Fig. 2E; Table 1). A full summarization of all the DEPs in the study can be found in Table 1.

## Discussion

Bile and BAs are critical in shaping the GIT microbiota, especially in chickens where current production feed models result in long periods of bile flow [28–32]. Microbial responses to bile and BAs impact host immune regulation and weight gain, yet proteomic responses of individual microbes to bile and BAs are not well understood [1, 2]. *Turicibacter bilis*, and other gut microbes, interact with bile and BAs, using them as an environmental cue for germination and biotransforming them in vitro [18, 33–35]. Here, we describe the growth and proteomic responses of *Turicibacter bilis* MMM721 to chicken bile and its main BA components: TCDCA and TCA.

MMM721 grown in BHIGL + Bile or BHIGL + TCDCA showed a reduction in CFUs and similar proteomic responses after 24 h. These similarities were not unexpected, as the most abundant BA in chickens is TCDCA [4, 11–13]. Previous studies and work from our lab demonstrated the ability of *Turicibacter*, including MMM721, to deconjugate taurine from TCDCA and TCA, forming CDCA and CA, respectively [35]. Unconjugated BAs more easily cross into the bacterial cell, though CDCA does so more efficiently, disrupting membrane integrity to a greater extent than CA, partially explaining the differences in growth and proteomic response [9, 28, 36].

Many DEPs in COG category J, translation, ribosomal structure, and biogenesis, were significantly decreased in BHIGL + Bile and BHIGL + TCDCA. Decreased protein synthesis would manifest as an overall decrease in bacterial growth, matching the observations in this and other studies [37–40]. COG category O, post-translational modification, protein turnover, and chaperones, DEPs were enriched in BHIGL + Bile when compared to BHIGL. Both BHIGL + Bile and BHIGL + TCDCA had molecular chaperones increased, likely in response to the ability of BAs to unfold cytoplasmic proteins, ensuring the proper folding of proteins within the cell [8, 41].

Only two DEPs were increased in all treatments. One was a peptidase dimerization domain protein that plays a protective role under BA stress [42, 43]. A d-alanine-d-alanine ligase involved in peptidoglycan biosynthesis was also increased, which may result from the disruptive effects BAs have on the bacterial cell surface [44, 45]. GMP synthase and an intersubunit bridge protein were decreased across all treatments. Subunit bridges are vital for the assembly and initiation of ribosomal translation, highlighting the decreased translational rate of MMM721 in response to BAs [46]. GMP synthase is responsible for the first step of de novo GMP synthesis. Decreased GMP synthase would reduce the GMP pool and potentially replication rate [47, 48].

The reduction in CFU/mL observed in the BHIGL + Bile and BHIGL + TCDCA suggests that stress response proteins observed in the proteomic data could be related to the stress the CDCA causes MMM721 or due to MMM721 cell death. However, studies show the half-life for bacterial proteins is approximately 20 h, so many of the DEPs observed in this study likely reflect the MMM721 response to TCDCA and whole chicken bile [49, 50]. Proteomic and transcriptomic analyses of earlier timepoints in the MMM721 growth cycle would be beneficial to more fully characterize the MMM721 response to TCDCA and whole bile.

The observed DEPs from the BHIGL + TCA treatment likely reflect the response of MMM721 to TCA. Of note, both a dehydrogenase (ONGNNMAF_01745) belonging to COG C (Energy production and conversion) and CplX (ONGNNMAF_00641), a subunit of an ATP-dependent Clp protease belonging to COG category O were significantly increased in BHIGL + TCA. CplX is an ATPase involved in several regulatory and proteolytic processes. *Lactobacillus delbrueckii* increases expression of CplX upon exposure to bile salts, suggesting this protein plays an important role in adaptation to BA stress [51, 52]. Dehydrogenases represent a broad family of enzymes that catalyze reduction reactions, with some classes, like hydroxysteroid dehydrogenases, capable of modifying the steroid ring of BAs themselves [53]. More work should be done to characterize the activity of the various MMM721 dehydrogenases. Many of the DEPs identified in this study were unable to be identified or had unknown functions. Further work characterizing the genome of MMM721 would be beneficial in future transcriptomic and proteomic studies.

## Conclusions

The present study constitutes the first characterization of the *Turicibacter bilis* response to chicken bile and individual bile acids using culturing and label-free LC–MS/MS strategies. Comparisons of the differentially expressed peptides were obtained from *Turicibacter bilis* MMM721 exposed to chicken bile, TCDCA, and TCA. Whole chicken bile and TCDCA both reduced MMM721 growth after 24 h of exposure, producing similar proteomic responses. Exposure of MMM721 to TCA did not substantially inhibit cell growth and up-regulated proteins involved in metabolism and post-translational modifications. Ultimately, the *Turicibacter bilis* MMM721 response to whole bile and bile acids is complex, involving proteins from several pathways. Understanding how members of the microbiota respond and modify BAs is critical to optimize animal nutrition and maintain efficient production.

## Limitations

Many of the DEPs have not yet been identified or characterized, making it difficult to determine responsive proteins and pathways and highlighting the importance of further characterization work for *Turicibacter bilis* MMM721. The 24 h exposure period may have coincided with the death phase of the MMM721 growth cycle, potentially exacerbating the decrease in CFU and the up- or down-regulation of various DEPs. The decrease in CFU associated with whole chicken bile and TCDCA might also be the result of MMM721 sporulating our entering into a viable but not culturable state, giving the perception of decreased CFU/mL. Additional analysis earlier in the MMM721 growth cycle would be informative.

## Supplementary Information

**Additional file 1.** OD_600_ growth curve comparing Turicibacter bilis growth in BHIGL broth supplemented with 0.1% whole avian bile (ATB), TCDCA (TCDCA), and TCA (TCA) compared to BHIGL-only (BHIGL). Error bars represent the standard error of the mean

## Data Availability

The data described in this Research Note can be accessed at MassIVE (https://massive.ucsd.edu/ProteoSAFe/static/massive.jsp) under the accession no. MSV000088421. The complete genome sequence for *Turicibacter bilis* MMM721 can be found in the National Center for Biotechnology Information (NCBI) GenBank database under the accession number CP071249.

## Abbreviations

TCDCA: Taurochenodeoxycholic acid
TCA: Taurocholic acid
GIT: Gastrointestinal tract
BAs: Bile acids
BHIGL: Brain heart infusion with lactate
PBS: Phosphate-buffered saline
ORFs: Open reading frames
DEPs: Differentially expressed proteins
COG: Clusters of orthologous groups
