# Supplementary figures and images for: Proteomic response of Turicibacter bilis MMM721 to chicken bile and its bile acids
Source: BMC Res Notes. 2022 Jul 2;15:236. doi: 10.1186/s13104-022-06127-8 (PMC9250206; doi:10.1186/s13104-022-06127-8)

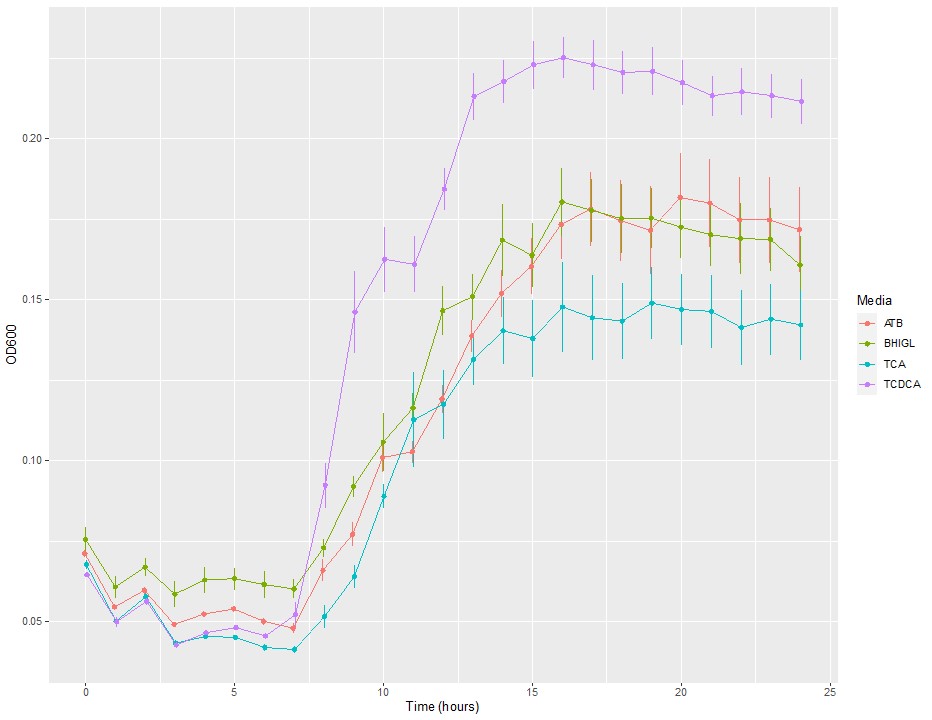

Supplement: Supplementary file 1 — Additional file 1. OD600 growth curve comparing Turicibacter bilis growth in BHIGL broth supplemented with 0.1% whole avian bile (ATB), TCDCA (TCDCA), and TCA (TCA) compared to BHIGL-only (BHIGL). Error bars represent the standard error of the mean [file 13104_2022_6127_MOESM1_ESM.jpg]
